# Optimized Solar-Simulated Photocatalysis of Congo Red Dye Using TiO_2_: Toward a Sustainable Water Treatment Approach

**DOI:** 10.3390/molecules30112388

**Published:** 2025-05-29

**Authors:** Davor Ljubas, Ante Vučemilović, Debora Briševac, Hrvoje Cajner, Hrvoje Juretić

**Affiliations:** 1 Faculty of Mechanical Engineering and Naval Architecture, University of Zagreb, 10000 Zagreb, Croatia; debora.brisevac@fsb.unizg.hr (D.B.); hrvoje.cajner@fsb.unizg.hr (H.C.); hrvoje.juretic@fsb.unizg.hr (H.J.); 2 The Department of Branch Tactics, Croatian Military Academy “Dr. Franjo Tuđman”, 10000 Zagreb, Croatia; ante.vucemilovic@suradnik.unizg.hr

**Keywords:** photocatalysis, titanium dioxide, Congo red dye, UV-A irradiation, central composite design

## Abstract

This study investigates a sustainable approach to the photocatalytic degradation of Congo red (CR) dye using titanium dioxide (TiO_2_) under simulated solar radiation, with a specific focus on the UV-A component of the radiation. The aim was to optimize reaction conditions to maximize dye removal efficiency while promoting environmentally friendly wastewater treatment practices. A central composite design (CCD) was implemented, and results were analyzed using analysis of variance (ANOVA). The key factors examined included TiO_2_ concentration, UV-A radiation intensity, CR dye concentration, and suspension depth. The optimal conditions determined were 222.37 mg/L TiO_2_, 20 W/m^2^ UV-A irradiation, 25 µmol/L CR dye concentration, and a suspension depth of 29 mm. Under these conditions, decolorization was achieved with the lowest absorbance (0.367 at 498 nm) and total organic carbon (0.805 mg/L) values, indicating effective dye degradation. The findings confirm that TiO_2_-assisted photocatalysis is a green and promising method for wastewater treatment. The potential use of natural solar radiation could reduce operational costs, making the process more sustainable. However, challenges such as photocatalyst recovery, aggregation, and the impact of the real wastewater matrices need further investigation.

## 1. Introduction

Dyes are known for their coloring effects and, unfortunately, their toxicity. Azo dyes, which contain azo aromatic groups, are widely used in industry and pose a significant environmental hazard due to their persistence and harmful effects on living organisms [1,2]. Therefore, it is very important to remove or degrade them from wastewater before releasing it into the environment. Congo red (CR) is a typical example of a synthetic anionic azo dye that finds application in the rubber, plastic, textile, paper, printing, and dyeing industries [3].

The toxicity of dyes is often associated with their mutagenic, carcinogenic, and teratogenic properties [4]. CR exhibits cytotoxic (genotoxic, hemotoxic, and neurotoxic), carcinogenic, and mutagenic effects, posing serious health risks, including potentially fatal outcomes at high concentrations.

CR affects the eyes, skin, and respiratory and reproductive systems. While CR itself is not always harmful, its reduction to aromatic amines increases its toxic effects. Benzidine, a toxic metabolite of CR, is a known bladder carcinogen that can cause allergic reactions and inhibit enzymatic activity by binding covalently to cellular macromolecules [1,5,6].

The lethal dose low (LDLo) refers to the smallest amount of a toxic substance that causes death in test animals under controlled conditions. This dosage is typically expressed in milligrams per kilogram of body weight and varies depending on the species studied. The substance can enter the body through ingestion, inhalation, or intravenous administration. The LDLo value of CR for humans is 143 mg/kg, indicating its toxicity. The harmful effects of azo dyes, including their genotoxic and cytotoxic properties, are also evident in aquatic plant life. When released into water, these dyes reduce light penetration, disrupting photosynthesis and negatively impacting the ecosystem [1,7].

Aquatic flora constitutes a main source of protein for the human diet. Thus, the consumption of aquatic animals known to have bioaccumulated CR can induce sicknesses like hypertension, fever, and cramps. Previous studies have assessed the toxicity of CR on the photosynthetic metabolism and growth of green algae, e.g., *Chlorella vulgaris*. Findings revealed that *Chlorella vulgaris* experienced a reduced rate of development, photosynthetic activity, and respiration [3].

Azo dyes exhibit significant resistance to biodegradation processes [6], making their removal or degradation crucial for environmental protection. The methods for removing azo dyes can be categorized into three main groups: (a) physical methods, such as adsorption, nanofiltration, ion exchange, and flocculation; (b) chemical methods, including H_2_O_2_-UV radiation, NaClO, O_3_, and others; and (c) biological degradation, encompassing both aerobic and anaerobic processes [8,9,10,11,12]. Despite their potential, biodegradation processes are often hindered by relatively low degradation efficiency [13].

Advanced oxidation processes (AOPs) are considered highly effective for wastewater treatment, as they enable the thorough degradation of contaminants without generating harmful by-products. These processes are driven by hydroxyl radicals (^•^OH), which can oxidize a wide spectrum of organic contaminants in a highly efficient and non-selective manner. Among AOPs, solar photocatalytic oxidation with semiconductor materials like titanium dioxide (TiO_2_) has garnered significant interest due to its promising performance [14,15,16,17].

While solar radiation alone does not provide sufficient energy for efficient dye degradation, its combination with a heterogeneous photocatalyst such as TiO_2_ greatly improves the degradation process. TiO_2_ absorbs UV radiation (wavelengths < 400 nm), which triggers electron excitation and leads to the formation of hydroxyl radicals, capable of breaking down organic pollutants [18,19,20]. Utilizing natural solar radiation to activate TiO_2_ also offers cost advantages over artificial UV sources, making it a more economically sustainable approach for wastewater treatment. In this study, we employed an artificial, lab-scale, solar-like lamp to evaluate the most suitable TiO_2_ crystalline structure and dosage for effective CR degradation.

The photocatalytic degradation efficiency and total organic carbon (TOC) removal were optimized using the Design-Expert 13.0. software. A central composite design (CCD) in the design of experiments was applied, extending the first-order model with additional measurement points at central and axial positions for enhanced model accuracy [21,22,23,24].

The primary objective of this research was to develop a predictive model, using the design of experiments methodology, that accurately describes the CR degradation process and optimizes key reaction parameters, including TiO_2_ concentration, UV-A irradiation intensity, CR dye concentration, and suspension depth. Optimization of photocatalysis is a crucial step to reach sustainable wastewater treatment using this AOP process [25,26]. The study’s novelty stems from its detailed, systematic optimization of the degradation process under simulated solar radiation, accounting for less commonly explored but practically crucial parameters such as suspension depth, thereby yielding directly applicable solutions for sustainable wastewater treatment. Through a systematic analysis of these factors, we aim to improve process efficiency while reducing the necessity for further experimental trials. In future work, we aim to employ advanced TiO_2_-based materials in this type of experiment, including those that (a) facilitate separation from suspensions, such as magnetic hybrid particles [18]; (b) incorporate modified nanocrystalline structures and dopants to enhance photocatalytic efficiency [27]; (c) are hybridized with graphitic carbon to achieve higher degradation rates of organic pollutants [28]; and (d) utilize various mixtures of anatase and rutile TiO_2_ to exploit their synergistic effects in degrading organic pollutants in water [29], among other possibilities. Furthermore, to address the challenge of photocatalyst recovery after the degradation process, particular attention should be given to the application of photocatalytic films, whose fixed configuration eliminates the need for post-treatment separation.

## 2. Results and Discussion

### 2.1. Irradiation Spectrum

**Figure 1:**
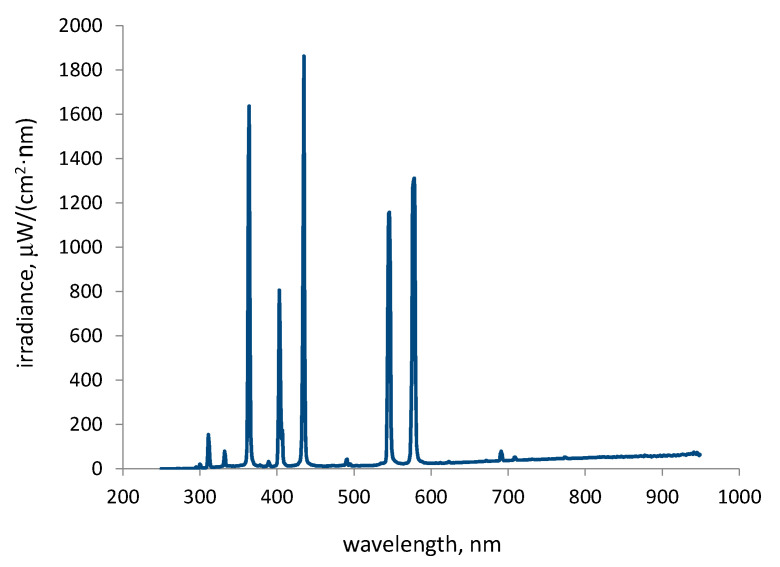
Overview of the irradiation spectrum of the OSRAM Ultra Vitalux 300 W lamp.

The irradiation spectrum of the solar-like lamp at the distance from the top of the reactor of 20 cm is shown in Figure 1, and the measured and calculated values for radiation in the most relevant spectral ranges are presented in Table 1.

As can be seen in Table 1, the chosen distance between the lamp and the solution surface in the reactor ensured the UV-A irradiance (which is important for activation of the photocatalyst) around two times higher than usually expected UV-A irradiance from the natural sunlight measured in the city of Zagreb in the time interval 10 a.m.–3 p.m. during summer months [30]. The higher irradiance was used with the intention to accelerate the degradation reactions of CR in aqueous solution, thereby enabling a faster optimization of the influential parameters.

Global radiation of the solar-like lamp was, at the distance of 20 cm, 1345.02 W/m^2^.

### 2.2. Absorbance and TOC

**Figure 2:**
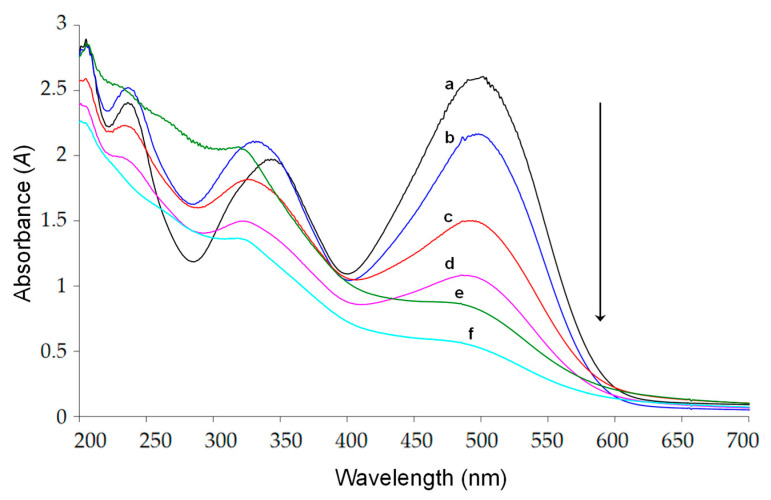
An example of the absorbance spectrum of Congo red before irradiation (**a**) and spectra of the irradiated solution (**b**–**f**).

The results of absorbance and *TOC* for each run are presented in Table 2, and the UV-VIS spectrum of the CR solution is in Figure 2. To ensure the most accurate representation of the responses (analytic results), they are presented as the ratio of the final (f) to the initial (0) value according to fractions for absorbance (*A*) and *TOC*:

$$\frac{A_{f}}{A_{0}},\;\frac{TOC_{f}}{TOC_{0}}.$$

An additional test was conducted to evaluate the influence of reducing the suspension volume by comparing two experiments: one using 70 mL and the other 100 mL of suspension in the reactor. The results showed negligible differences, leading to the conclusion that the effect of volume reduction during sampling is also negligible.

As presented in Table 2, the absorbance parameter *A* exhibits more pronounced changes within the first 30 min compared to *TOC*. This can be attributed to the fact that absorbance measurements capture structural transformations of the parent compound (degradation), whereas *TOC* reflects only the extent of complete mineralization—specifically, the portion of the organic compound that has been fully oxidized and released as CO_2_ from the solution (total removal from water).

### 2.3. Results of ANOVA 2FI Model

The results of the analysis of variance (ANOVA) presented in Table 3 provide insights into the statistical significance model.

The F-test value of 34.74 indicates that the model is statistically significant, with less than a 0.01% probability that this high value occurs due to random noise. Furthermore, all variables with a *p*-value lower than 0.0500 are considered statistically significant. The significant variables are A (TiO_2_ concentration), B (UV-A intensity), C (CR concentration), D (suspension depth), and the interaction AB (TiO_2_ × UV-A). Other variables with *p*-values greater than 0.1000 are not statistically significant; however, they contribute to the model’s hierarchy and are therefore retained.

The F-test value of 10.22 in Table 4 indicates that the model is statistically significant, with less than a 0.01% probability that this high value could be caused by noise. The variables A, B, C, AD, BD, CD, and C^2^ are statistically significant.

The regression equation for absorbance is:

Absorbance = 0.80541 + 8.750738 × 10^−5^ × A − 0.02202 × B + 0.01426 × C − 0.01763 × D − 3.45184 × 10^−5^ × AB + 0.00091 × BD (1)

while the regression equation for *TOC* is:

*TOC* = 2.36159 + 0.00259 × A − 0.03570 × B − 0.05031 × C − 0.03820 × D − 0.00014 × AD + 0.00133 × BD + 0.00114 × CD + 0.00041 × C^2^ (2)

### 2.4. Model Diagnostics —Graphs

**Figure 3:**
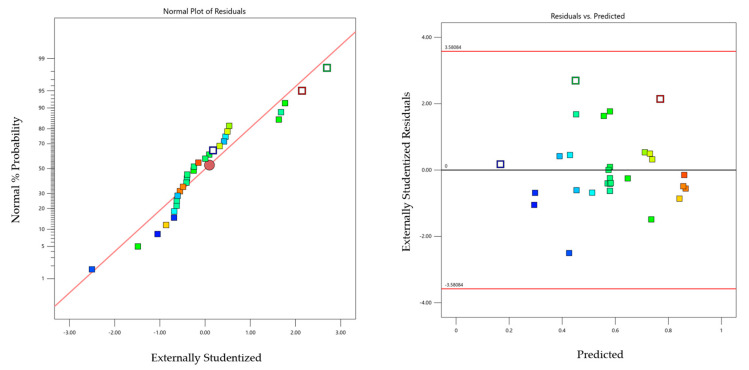
Graphical analysis of residuals in the case of the variable absorbance.

The normal probability and studentized residual graphs are presented in Figure 3. The studentized residuals indicate how well the actual and predicted standard deviations of the results overlap.

Residual analysis for the *TOC* variable exhibited similar features.

**Figure 4:**
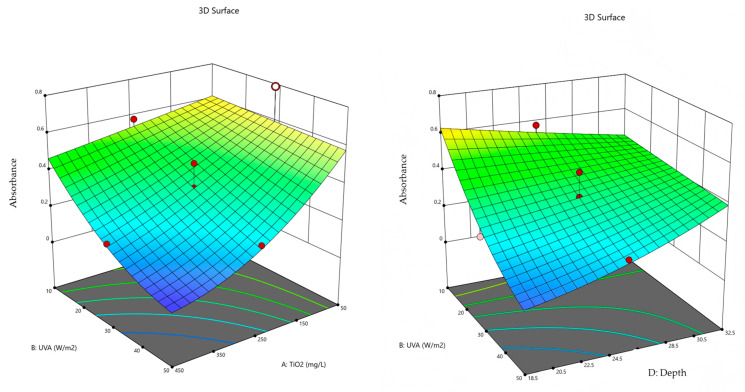
Response surface in relation to absorbance *A*.

**Figure 5:**
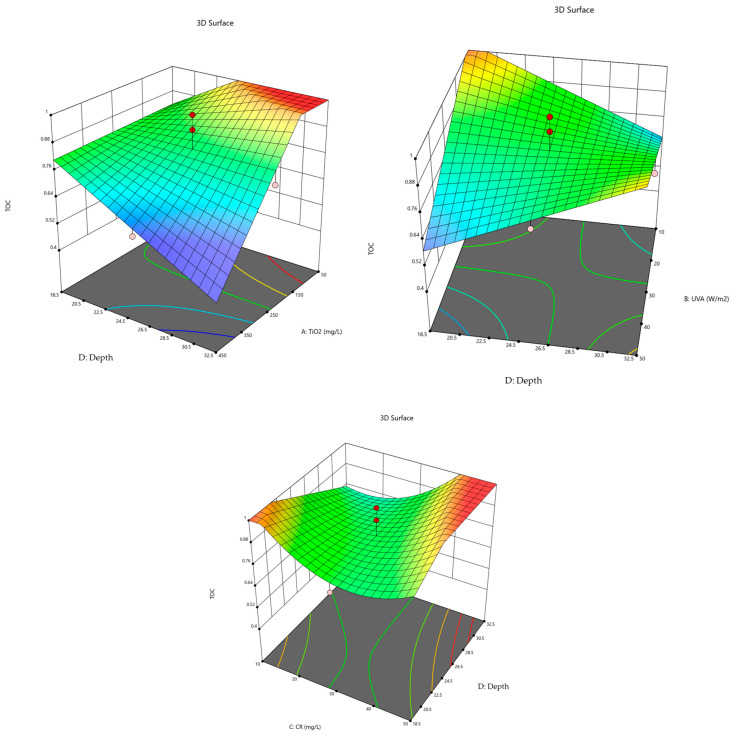
Response surface in relation to *TOC*.

Response Surface Analysis: the response surfaces for the degradation of CR dye are presented in Figure 4 and Figure 5.

In Figure 4 (left), the dependence of absorbance on the concentration of the TiO_2_ photocatalyst and the intensity of UV irradiation is shown for a CR dye concentration of 30 µmol/L and a suspension depth of 25.5 mm in the reactor. The absorbance value increases with a lower amount of photocatalyst and lower irradiation intensity, resulting in reduced dye degradation. The best degradation is achieved at the lowest absorbance value, which is expected at the highest photocatalyst concentration and the strongest UV-A irradiation. In Figure 4 (right), the dependence of absorbance on irradiation intensity and suspension depth in the reactor is presented for a photocatalyst concentration of 250 mg/L and irradiation of 30 W/m^2^. It is evident that absorbance reaches its lowest values at the highest irradiation intensity and the smallest suspension depth.

In Figure 5 (top left), the dependence of *TOC* values on the concentration of the TiO_2_ photocatalyst and suspension depth is shown for a CR dye concentration of 30 µmol/L and an irradiation intensity of 30 W/m^2^. The *TOC* value increases with lower photocatalyst concentration and greater suspension depth, resulting in reduced dye degradation efficiency. The best degradation efficiency is achieved at the lowest *TOC* value, which corresponds to the highest photocatalyst concentration and shallowest suspension.

In Figure 5 (top right), the dependence of *TOC* values on irradiation intensity and suspension depth in the reactor is shown for a photocatalyst concentration of 250 mg/L and a CR dye concentration of 30 µmol/L. It is evident that *TOC* reaches its lowest values at the highest irradiation intensity and the smallest suspension depth.

The dependence of *TOC* values on CR dye concentration and suspension depth in the reactor is shown in Figure 5 (bottom) for a photocatalyst concentration of 250 mg/L and an irradiation intensity of 30 W/m^2^. It is observed that *TOC* reaches its lowest values at the lowest CR dye concentration and the highest suspension depth, indicating that a low pollutant load combined with optimized geometry enhances mineralization.

### 2.5. Conditions for Optimization

Table 5 describes the conditions for the optimization of the photocatalytic degradation of CR dye, while Table 6 presents the optimal factor values and their alignment with the desired conditions.

At the optimized factor values of TiO_2_ concentration of 222.37 mg/L, UV-A irradiation intensity of 20 W/m^2^, CR dye concentration of 25 µg/L, and a suspension depth of 29 mm, the best degradation of CR dye and the lowest *TOC* value are achieved, which still require further validation through confirmatory experiments (control run).

**Figure 6:**
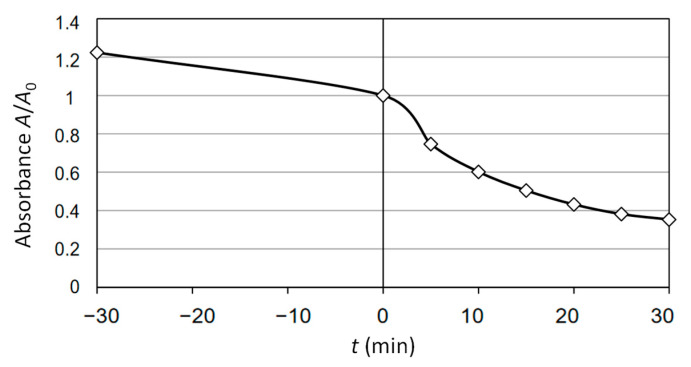
Control run with selected parameters from Table 6.

The outcome of the control run is presented in Figure 6, where the final absorbance ratio (A₃₀/A₀) was 0.3533, differing from the predicted value of 0.367 by 3.75%. The time period marked as −30 to 0 min was a period of the adsorption process (stirring “in the dark”).

The experimental results showed that the TiO_2_ concentration and UV-A irradiation intensity significantly influenced the extent of CR dye degradation. ANOVA results indicated that factors A (TiO_2_), B (UV-A), C (CR), and D (suspension depth) were significant in the model (*p* < 0.05).

Response surface analysis revealed that optimal CR dye degradation requires higher TiO_2_ concentrations and stronger UV irradiation, while excessive TiO_2_ concentrations can lead to agglomeration, reducing its active surface area and degradation efficiency. On the other hand, increasing suspension depth resulted in lower degradation efficiency due to reduced light penetration.

The design of experiments (DOE) and ANOVA, combined with a lamp with the radiation wavelengths that can be found in natural solar radiation, show a key sustainability advantage of this approach—its ability to harness natural solar energy, particularly in the UV-A spectrum, leading to substantial reductions in energy consumption and associated environmental impacts relative to artificial illumination. In addition, the process demonstrates a high potential for achieving appropriate mineralization of organic contaminants, positioning it as a promising candidate for large-scale wastewater treatment applications.

### 2.6. Comparison of Azo Dye Treatment Methods in Wastewaters

To highlight the potential of applying this method—photocatalytic treatment utilizing solar radiation—a comparative overview of five applicable techniques is presented in Table 7. These methods are considered for the treatment and degradation of azo dyes in wastewater streams prior to their discharge into the environment.

As illustrated, each treatment method has inherent advantages and limitations. Therefore, combining complementary techniques can lead to more efficient and environmentally sustainable solutions. One particularly promising approach is the integration of membrane separation with photocatalysis, whereby photocatalysis is utilized to degrade concentrated effluents, ideally harnessing natural solar radiation during optimal periods. This synergy maximizes treatment efficiency while minimizing environmental impact.

## 3. Materials and Methods

### 3.1. Methods

**Figure 7:**
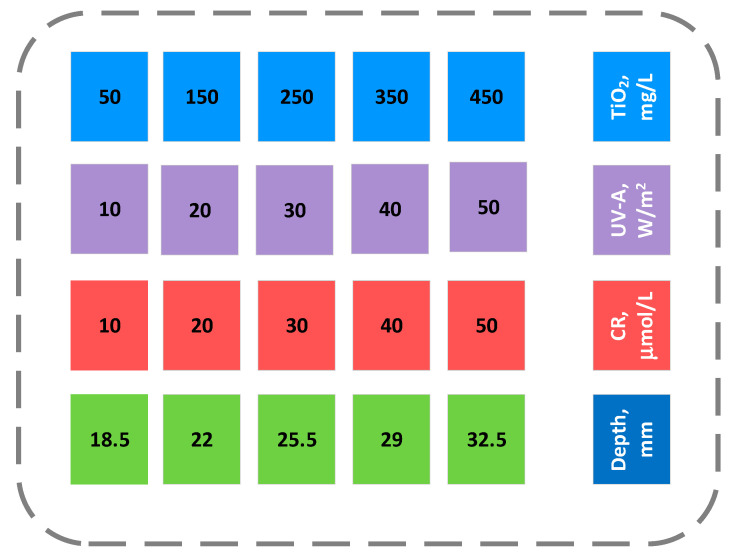
Overview of independent variables’ variation in experiments.

For the purpose of the study, 30 experiments were performed, and their results are presented in Table 8. The table includes independent variables, i.e., influencing factors such as TiO_2_ concentration, UV-A intensity, CR concentration, and suspension depth, along with the photocatalytic degradation (measured as absorbance A (498)) and *TOC* as response variables. Variation in the independent variables is presented in Figure 7.

The experiments were conducted in a randomized order, while the central value of the CCD model was repeated six times to determine the pure error. The efficiency of CR dye removal from the suspension was assessed by response analysis within the model.

To determine the interactions among variables and their impact on responses, analysis of variance (ANOVA) was employed. The model’s fit was evaluated using the coefficients of determination (R^2^ and adjusted R^2^), while statistical significance was verified through adequate precision values and the F-test.

### 3.2. Materials

The TiO_2_ powder used in the experiments was Degussa P-25 (currently marketed by Evonik Co.), a well-characterized material with well-established properties. The P-25 TiO_2_ had a purity of 99.9% and was used as received, without any further modification. It consists primarily of the anatase phase (75–80%) with a rutile content of 20–25% and has a specific surface area (BET) of 50–54 m^2^/g, corresponding to a mean particle size of approximately 30 nm. The pore volume is 0.196 cm^3^/g, and the band gap energy (*E*_g_) is 3.15 eV [32,33].

CR dye was supplied by ACROS Co. as a high-purity biological stain (90%) and used as a model compound without further purification.

The samples were filtered by vacuum filtration through 0.45 μm cellulose nitrate membranes provided by Sartorius Co. (Göttingen, Germany). All the chemicals and materials were used as received, without further purification.

### 3.3. Experimental Section

For the experiment, a 500 mL aqueous solution of CR dye was prepared. The solution was stirred in the dark for 24 h and subsequently filtered through a 0.45 µm pore size membrane filter with a diameter of 47 mm (Lobex Ltd. MCE Filter-Bio). A 250 mL aliquot of the solution, containing varying CR dye concentrations (10–50 µmol/L), was transferred to a glass reactor, followed by the addition of TiO_2_ at different concentrations (50, 150, 250, 350, and 450 mg/L). The experiments were conducted in glass reactors with depths ranging from 18.5 to 32.5 mm and with the stirring speed of approximately 120 rpm. During the experiments, samples were collected at intervals of 5 to 30 min and analyzed using a UV-VIS spectrophotometer at a wavelength of 498 nm. Additionally, total organic carbon (TOC) was measured as an indicator of the degree of CR dye mineralization.

**Figure 8:**
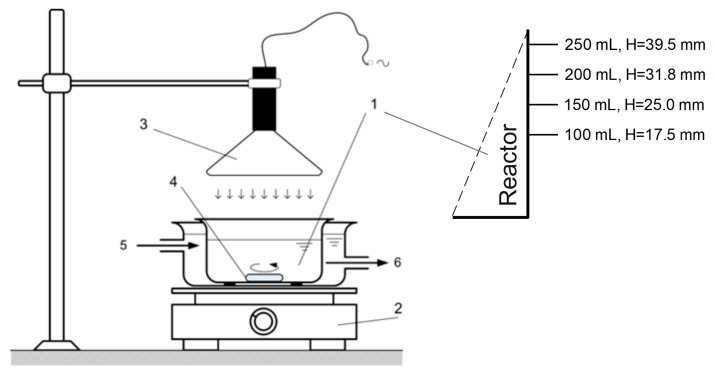
Schematic diagram of photoreaction apparatus. 1. reactor, 2. magnetic agitator, 3. UV-VIS-IR lamp (“solar like” lamp), 4. stirring bar, 5. water cooling inlet, 6. water cooling outlet.

The experimental setup with the UV-VIS-IR lamp is shown in Figure 8. The lamp’s radiation components correspond to parts of the natural solar spectrum, as illustrated in Figure 1. Although this lamp is not a solar simulator, throughout the text we will refer to it as a lamp simulating solar radiation, since it provides artificial radiation that partially resembles solar light.

The lamp employed in this study was the Ultra Vitalux 300W model, manufactured by Osram Co (Munich, Germany).

After homogenizing the suspension in an ultrasonic bath for 5 min, a sample was taken for TOC measurement. Prior to exposing the reactor to UV light, the solution was stirred in the dark on a magnetic stirrer for 30 min to establish adsorption–desorption equilibrium. The reactor was then placed on a magnetic stirrer under a UV lamp at varying distances (22, 25, 30, 40, and 55 cm) to achieve different radiation intensities (10, 20, 30, 40, and 50 W/m^2^). Additionally, two distinct glass reactors were used to accommodate different solution depths (18.5, 22, 25.5, 29, and 32.5 mm).

Samples of 5 mL were collected using a syringe at time intervals of 5, 10, 15, 20, 25, and 30 min and filtered through a 0.45 µm membrane filter with a 25 mm diameter. The filtered samples were analyzed using a UV-VIS spectrophotometer (type 8453, manufactured by Hewlett Packard Co.; Palo Alto, USA) to determine CR absorbance at 498 nm. At the end of the experiment (after 30 min), an additional sample was taken for TOC measurement. TOC analysis was performed using a Shimadzu TOC-VCPH analyzer. The sample volume was 20 mL, and the NPOC (non-purgeable organic carbon) method was applied.

The pH value of the solution was measured both before UV exposure and at the end of the experiment.

## 4. Conclusions

This study demonstrated the effectiveness of TiO_2_-assisted photocatalysis under artificial solar-like UV-A irradiation for the degradation of Congo red (CR) dye in aqueous solutions. Using a central composite design (CCD) and analysis of variance (ANOVA), we optimized reaction conditions to achieve maximum dye degradation efficiency. The results suggest that TiO_2_ photocatalysis is a promising method for treating dye-contaminated wastewater, providing an environmentally sustainable approach to pollution control. By optimizing key operational parameters, high removal efficiency and significant mineralization were achieved, indicating the potential of this process for practical wastewater treatment applications. A key strength of this study lies in the integration of Design of Experiments (DOE) methodology with photocatalytic treatment to identify optimal parameters among four of them.

Key parameters influencing degradation efficiency include TiO_2_ concentration, UV-A intensity, CR dye concentration, and suspension depth. Response surface methodology (RSM) confirmed the significance of these factors, with optimal conditions identified as 222.37 mg/L TiO_2_, 20 W/m^2^ UV-A irradiation, 25 µg/L CR dye, and a suspension depth of 29 mm. Under these conditions, the lowest absorbance at 498 nm (0.367) and TOC (0.805 mg/L) values were achieved, indicating effective CR dye removal.

Although this study successfully demonstrated CR dye degradation, some limitations should be addressed in future research. TiO_2_ nanoparticle aggregation at higher concentrations can reduce its photocatalytic efficiency, necessitating strategies for improved catalyst dispersion. Additionally, real wastewater contains diverse contaminants that may affect photocatalytic performance, requiring further optimization under complex environmental conditions.

One of the major sustainability benefits of this method is the potential to utilize natural solar radiation, particularly its UV-A component, which can dramatically reduce operational energy costs and environmental impact compared to artificial light sources. Furthermore, the method enables near-complete mineralization of organic pollutants, making it an attractive alternative for large-scale wastewater treatment. However, further research is needed to assess long-term photocatalyst stability, reusability, and scalability for real wastewater treatment systems.

## Figures and Tables

**Table 1:** Measured and calculated (integrated) values of the irradiance at 20 cm distance from the lamp.

Irradiation Range	Total Irradiance in the Range, W/m^2^
UV C: 250–289 nm	0.173
UV B: 290–319 nm	4.92
UV A: 320–399 nm	56.63
VIS: 400–750 nm	268.20

IR radiation was not measured because it does not possess sufficient energy to initiate the photocatalytic process (or photolytic process).

**Table 2:** Experimental values *A*(498) and *TOC* obtained after each test run. *A*(498)_0_ and *TOC*_0_ represent the initial values after filtering the CR solution through a 0.45 µm membrane, and *A*_30_ and *TOC*_30_ represent values of the parameters after 30 min of irradiation.

Run	*A*(498)_0_	*TOC*_0_, mg/L	*A*_30_/*A*_0_	*TOC*_30_/*TOC*_0_
1	0.51833	5.007	0.09241	0.922
2	0.72360	6.198	0.20830	0.747
3	0.76529	9.047	0.39899	0.761
4	0.78161	8.353	0.33588	0.986
5	0.83306	8.689	0.34327	0.903
6	1.2368	12.875	0.63190	1.000
7	1.2215	8.792	0.54837	0.845
8	0.40222	3.024	0.03135	0.686
9	1.0387	6.873	0.78627	1.000
10	0.52965	4.842	0.41176	1.000
11	0.79584	6.371	0.22289	0.763
12	0.49248	3.535	0.30121	0.853
13	1.4572	9.955	0.69174	1.000
14	0.70445	5.220	0.30540	0.783
15	0.70198	4.989	0.17247	0.581
16	0.78837	5.689	0.31761	0.791
17	0.94556	7.634	0.57289	0.819
18	0.80515	5.957	0.29005	0.781
19	0.07364	0.975	0.05423	1.000
20	1.0348	7.361	0.31514	0.867
21	0.5229	4.478	0.33033	0.883
22	0.73002	6.207	0.29018	0.968
23	1.0598	8.107	0.43202	0.856
24	0.3419	3.813	0.06777	0.632
25	0.34969	4.371	0.17695	0.581
26	1.4261	10.4515	0.72512	1.000
27	0.82627	9.160	0.47506	0.806
28	0.86167	8.704	0.57740	0.816
29	1.2639	9.635	0.68785	1.000
30	0.95448	8.572	0.291583	0.739

**Table 3:** Results of ANOVA 2FI model for absorbance.

Source	Sum of Squares	df	Mean Square	F-Value	*p*-Value	
Model	0.9019	6	0.1503	34.74	<0.0001	significant
A-TiO_2_	0.2157	1	0.2157	49.85	<0.0001	
B-UVA	0.1359	1	0.1359	31.40	<0.0001	
C-CR	0.4881	1	0.4881	112.80	<0.0001	
D-Depth	0.0270	1	0.0270	6.23	0.0201	
AB	0.0191	1	0.0191	4.41	0.0470	
BD	0.0161	1	0.0161	3.73	0.0660	
Residual	0.0995	23	0.0043			
Lack of Fit	0.0831	18	0.0046	1.41	0.3761	not significant
Pure Error	0.0164	5	0.0033			
Cor Total	1.00	29				

**Table 4:** Results of ANOVA 2FI reduced squared model for *TOC*.

Source	Sum of Squares	df	Mean Square	F-Value	*p*-Value	
Model	0.4382	8	0.0548	10.22	<0.0001	significant
A-TiO_2_	0.2520	1	0.2520	47.00	<0.0001	
B-UVA	0.0069	1	0.0069	1.29	0.2696	
C-CR	0.0242	1	0.0242	4.51	0.0463	
D-Depth	0.0001	1	0.0001	0.0232	0.8806	
AD	0.0394	1	0.0394	7.35	0.0135	
BD	0.0348	1	0.0348	6.50	0.0191	
CD	0.0256	1	0.0256	4.78	0.0409	
C^2^	0.0275	1	0.0275	5.13	0.0348	
Residual	0.1073	20	0.0054			
Lack of Fit	0.0767	15	0.0051	0.8383	0.6412	not significant

**Table 5:** Conditions for the optimization of CR degradation.

Name	Goal	Lower Limit	Upper Limit	Lower Weight	Upper Weight	Importance
A:TiO_2_	minimize	150	350	1	1	3
B:UVA	minimize	20	40	1	1	3
C:CR	is target = 25	20	40	1	1	3
D:Depth	is in range	22	29	1	1	3
Absorbance	minimize	0.031356	0.786271	1	1	3
TOC	minimize	0.581512	1.07353	1	1	3

**Table 6:** Optimal factor values.

Number	TiO_2_	UV-A	CR	Depth	Absorbance	TOC	Desirability	
1	222.372	20.000	25.000	29.000	0.367	0.805	0.674	Selected
2	222.075	20.000	24.997	29.000	0.367	0.806	0.674	
3	218.487	20.000	25.000	29.000	0.370	0.811	0.674	
4	226.254	20.000	25.000	29.000	0.364	0.799	0.674	
5	216.159	20.000	25.000	29.000	0.371	0.815	0.674	

**Table 7:** Comparative analysis of water treatment methods in use today [31].

Water Treatment Method	Advantages and Positive Aspects	Drawbacks and Limitations
Adsorption (on granulated or powdered activated carbon)	Provides effective dye removal from wastewater; the treatment is easy to implement and represents a well-established and widely adopted approach.	Efficiency depends on the properties of the adsorbent and environmental conditions; there is a risk of secondary pollution generation.
Membrane Separation (ultrafiltration, nanofiltration, reverse osmosis)	Capable of removing various dye types, allows selective separation, and occupies a relatively limited spatial footprint.	Installation and operational costs are high; it requires specific conditions such as pressurization; it is susceptible to clogging and has a limited operational lifespan; and there is a problem with concentrated flow with pollutants.
Coagulation and flocculation	A straightforward process requiring low-cost equipment; especially effective for hydrophobic dyes.	Generates significant amounts of sludge that are difficult to manage; less suitable for hydrophilic dye removal, equipment material can easily corrode.
Photocatalysis	Efficient in degrading persistent dye molecules, enabling complete breakdown; characterized by high energy efficiency if natural solar radiation is used	Separation of photocatalyst; if the process uses suspended particles, intermediate products can be more toxic after treatment than the parent product
Biological treatment (biodegradation by microorganisms)	Enables selective biodegradation via microorganisms, with minimal ecological toxicity.	Ineffective for high-concentration dye wastewaters or those with poor light penetration; slow process dependent on specific environmental conditions.

**Table 8:** Experimental values for photocatalytic tests.

Run	TiO_2_, mg/L	UV-A, W/m^2^	CR, μmol/L	Depth, mm
1	150	40	20	22
2	250	50	30	25.5
3	250	30	30	32.5
4	350	20	20	22
5	250	30	30	25.5
6	150	40	40	29
7	150	40	40	22
8	350	40	20	22
9	50	30	30	25.5
10	150	40	20	29
11	250	30	30	18.5
12	150	20	20	22
13	250	30	50	25.5
14	250	30	30	25.5
15	450	30	30	25.5
16	250	30	30	25.5
17	350	20	40	29
18	250	30	30	25.5
19	250	30	10	25.5
20	350	40	40	29
21	150	20	20	29
22	250	30	30	25.5
23	350	20	40	22
24	350	40	20	29
25	350	20	20	29
26	150	20	40	29
27	250	30	30	25.5
28	250	10	30	25.5
29	150	20	40	22
30	350	40	40	22

## Data Availability

Data can be provided upon request.

## References

[1] Siddiqui S.I., Allehyani E.S., Al-Harbi S.A., Hasan Z., Abomuti M.A., Rajor H.K., Oh S. (2023). Investigation of Congo Red Toxicity towards Different Living Organisms: A Review. Processes.

[2] Hoang N.T.T., Nguyen D.D. (2023). Improving the Degradation Kinetics of Industrial Dyes with Chitosan/TiO_2_/Glycerol Films for the Sustainable Recovery of Chitosan from Waste Streams. Sustainability.

[3] Oladoye P.O., Bamigboye M.O., Ogunbiyi O.D., Akano M.T. (2022). Toxicity and decontamination strategies of Congo red dye. Groundw. Sustain. Dev..

[4] Periyasamy A.P. (2024). Recent Advances in the Remediation of Textile-Dye-Containing Wastewater: Prioritizing Human Health and Sustainable Wastewater Treatment. Sustainability.

[5] Lellis B., Fávaro-Polonio C.Z., Pamphile J.A., Polonio J.C. (2019). Effects of textile dyes on health and the environment and bioremediation potential of living organisms. Biotechnol. Res. Innov..

[6] Naseem K., Farooqi Z.H., Begum R., Irfan A. (2018). Removal of Congo red dye from aqueous medium by its catalytic reduction using sodium borohydride in the presence of various inorganic nano-catalysts: A review. J. Clean. Prod..

[7] Imran M., Shaharoona B., Crowley D., Khalid A., Hussain S., Arshad M. (2015). The stability of textile azo dyes in soil and their impact on microbial phospholipid fatty acid profiles. Ecotoxicol. Environ. Saf..

[8] Muruganandham M., Swaminathan M. (2004). Solar photocatalytic degradation of a reactive azo dye in TiO_2_-suspension. Sol. Energy Mater. Sol. Cells.

[9] Bouberka Z., Kacha S., Kameche M., Elmaleh S., Derriche Z. (2005). Sorption study of an acid dye from aqueous solutions using modified clays. J. Hazard. Mater..

[10] Scott J.P., Ollis D.F. (1995). Integration of chemical and biological oxidation process for water treatment: Review and recommendation. Environ. Prog..

[11] Amagan B., Turan M., Celike M. (2004). Equilibrium studies on the adsorption of reactive azo dyes into zeolite. Desalination.

[12] Hashemian D. (2007). Study of adsorption of acid dye from aqueous solutions using bentonite. Main Group Chem..

[13] Purkait M.K., Maiti A., DasGupta S., De S. (2007). Removal of congo red using activated carbon and its regeneration. J. Hazard. Mater..

[14] Dai S., Zhuang Y., Chen Y., Chen L. (1995). Study on the relationship between structure of synthetic organic chemicals and their biodegradability. Environ. Chem..

[15] Legrini O., Oliveros E., Braun A.M. (1993). Photochemical Processes for Water Treatment. Chem. Rev..

[16] Goslich R., Dillert R., Bahnemann D. (1997). Solar water treatment: Principles and reactors. Water Sci. Technol..

[17] Vidal A. (1998). Developments in solar photocatalysis for water purification. Chemosphere.

[18] Ljubas D., Franzreb M., Hansen H.C.B., Weidler P.G. (2014). Magnetizing of nano-materials on example of Degussa’s P-25 TiO_2_ photocatalyst: Synthesis of magnetic aggregates, characterization and possible use. Sep. Purif. Technol..

[19] Ljubas D. (2005). Solar photocatalysis-a possible step in drinking water treatment. Energy.

[20] Linsebigler A.L., Lu G., Yates J.T. (1995). Photocatalysis on TiO_2_ surfaces: Principles, Mechanisms and selected results. Chem. Rev..

[21] Serpone N., Sauvé G., Koch R., Tahiri H., Pichat P., Piccinini P., Pelizzeti E., Hidaka H. (1996). Standardization protocol of process efficiencies and activation parameters in heterogeneous photocatalysis: Relative photonic efficiencies ζ_r_. J. Photochem. Photobiol. A.

[22] Ponnusamy S.K., Subramarian R. (2013). Process optimization studies of Congo red dye adsorption onto cashew nut shell using response surface methodology. Int. J. Ind. Chem..

[23] Cho H., Zoh K.D. (2007). Photocatalytic degradation of azo dye (Reactive Red 120) in TiO_2_/UV system: Optimization and modeling using a response surface methodology (RSM) based on the central composite desig. Dyes Pigment..

[24] Fathinia M., Khataee A.R., Zarei M., Aber S. (2010). Comparative Photocatalytic Degradation of Two Dyes on Immobilized TiO_2_ Nanoparticles: Effect of Dye Molecular Structure and Response Surface Approach. J. Mol. Catal. A.

[25] Ngulube K.F., Abdelhaleem A., Fujii M., Nasr M. (2024). Synergism of Artificial Intelligence and Techno-Economic for Sustainable Treatment of Methylene Blue Dye-Containing Wastewater by Photocatalysis. Sustainability.

[26] Korbahti K., Rauf M.A. (2008). Application of Response Surface Analysis to the Photolytic Degradation of Basic Red 2 Dye. Chem. Eng. J..

[27] Zhang Z., Wang C.C., Zakaria R., Ying J.Y. (1998). Role of Particle Size in Nanocrystalline TiO_2_-Based Photocatalysts. J. Phys. Chem. B.

[28] Sanchez Tobon C., Panžić I., Bafti A., Matijašić G., Ljubas D., Ćurković L. (2022). Rapid Microwave-Assisted Synthesis of N/TiO_2_/rGO Nanoparticles for the Photocatalytic Degradation of Pharmaceuticals. Nanomaterials.

[29] Guimarães R.R., Parussulo A.L.A., Toma H.E., Araki K. (2016). Enlightening the synergic effect of anatase/rutile mixtures in solar cells. Electrochim. Acta.

[30] Kulišić P. (1991). New Energy Sources: Solar Energy and Wind Energy (Novi Izvori Energije: Sunčana Energija i Energija Vjetra).

[31] Guo J., Zhou T., Guo H., Ge C., Lu J. (2025). Application of nano-TiO_2_@adsorbent composites in the treatment of dye wastewater: A review. J. Eng. Fibers Fabr..

[32] Sanchez Tobon C., Ljubas D., Mandić V., Panžić I., Matijašić G., Ćurković L. (2022). Microwave-Assisted Synthesis of N/TiO_2_ Nanoparticles for Photocatalysis under Different Irradiation Spectra. Nanomaterials.

[33] Patil S.R., Akpan U.G., Hameed S.K., Samdarshi S.K. (2012). A comparative study of the photocatalytic efficiency of Degussa P25, Qualigens, and Hombikat UV-100 in the degradation kinetic of congo red dye. Desalination Water Treat..

